# Microvascular Parameters Help to Predict Stroke Risk in the Asian Diabetic Population in Taiwan: A Population Based Case-Control Study

**DOI:** 10.3389/fneur.2018.00719

**Published:** 2018-08-29

**Authors:** Sui-Foon Lo, Wei-Liang Chen, Chih-Hsin Muo, Pei-Chun Chen, Shih-Yin Chen, Chih Lan Kuo, Fung-Chang Sung

**Affiliations:** ^1^Department of Physical Medicine and Rehabilitation, China Medical University Hospital, Taichung, Taiwan; ^2^Department of Chinese Medicine, College of Chinese Medicine, China Medical University, Taichung, Taiwan; ^3^Medical College of Wisconsin and Children's Hospital of Wisconsin, Milwaukee, WI, United States; ^4^Management Office for Health Data, China Medical University Hospital, China Medical University, Taichung, Taiwan; ^5^Department of Public Health, College of Public Health, China Medical University, Taichung, Taiwan; ^6^Department of Medical Genetics, China Medical University Hospital, Taichung, Taiwan; ^7^Department of Health Services Administration, College of Public Health, China Medical University, Taichung, Taiwan

**Keywords:** case-control study, diabetes, microvascular complications, ischemic stroke, hemorrhagic stroke, insurance data

## Abstract

Intensive glycemic control has not shown consistent findings in stroke prevention for diabetes patients, particularly for those with microvascular complications. This case-control study evaluates the risks of stroke in Asian diabetic population with microvascular complications. From the insurance claims of Taiwan, we identified 67,426 type 2 diabetic mellitus (DM) patients with newly diagnosed stroke in 2000–2011 and 134,852 randomly selected controls with DM but without stroke, matched by sex, age, and number of years since diagnosis of DM. Conditional logistic regression analysis measured crude odds ratios (OR) and adjusted odds ratio (aOR) of stroke and 95% confidence intervals (CI) for associations with demographic status, comorbidities, and microvascular complications: retinopathy (RetP), neuropathy (NeuP) or nephropathy (NepP). The aOR of stroke increased significantly associated with each complication: 1.47 with RetP, 1.73 with NeuP and 1.23 with NepP. The risk increased further when there was a combination of complications. The overall aOR of stroke was 2.83 (95% CI 2.58–3.09) for stroke patients with 3 microvascular complications. The corresponding aOR of ischemic stroke was 2.64 (95% CI 2.39–2.91) and that of hemorrhagic stroke was 4.12 (95% CI 3.25–5.22). The number of microvascular complications positively correlated to the prevalence of comorbidity (*p* < 0.01). This study suggests that microvascular complications are significant stroke predictors, with a greater involvement for ischemic stroke than for hemorrhagic stroke. Multiple microvascular complications interactively increase the stroke risk. Our study contributes to the identification of high-risk subjects for stroke prevention and adequate glycemic control.

## Introduction

Stroke is a well-known long-term macrovascular complication of type 2 diabetes, in addition to hypertension. Studies revealed a reduced risk of cardiovascular events for diabetic mellitus (DM) patients with improved glycemic control (1). Therefore, effort has been taken to enhance stroke prevention by intensive glycemic control. Modern large randomized control trials have shown some convincing results in terms of reducing cardiovascular risk (2–4), but there is no clear beneficial effect on stroke prevention (5, 6). Furthermore, the Action to Control Cardiovascular Risk in Diabetes (ACCORD) trial found that subjects with the intensive glycemic control have significant higher mortality than those with conventional glycemic control (5). In response to those results, current guideline suggests less intensive glycemic control to avoid mortality (7).

Due to the mixed results of intensive glycemic control and the complication of that, subgroup analysis was performed to identify high risk population in which intensive glycemic control can produce more convincing effects. For example, Veterans Affairs Diabetes Trial (VADT) suggested more beneficial effect of intensive glycemic control in patients with DM less than 15 years (8). Comparing among different trials, it has been noticed that studies recruiting younger population with newly diagnosed DM tend to have better outcome (3, 4), indicting younger population might have better benefit-risk ratio with intensive glycemic control. This heterogeneity is also noticed when it comes to the mortality, high glycemic index is associated with high mortality in intensive glycemic control in ACCORD trial (9). Taken together, intensive glycemic control might be only beneficial in predefined subgroups (10).

Opposed to the cardiovascular events, microvascular complications can be significantly reduced after intensive glycemic control (6) and might be a better indicator of the overall vascular healthiness. It has been postulated that both microvascular complications and cardiovascular events share similar risk factors and underlying pathological changes (11). For example, endothelial dysfunction in diabetic patients has been considered to be an underlying cause for diabetic vasculopathy (12). A recent meta-analysis has revealed that diabetic retinopathy and microalbuminuria could increase the risk of cardiovascular events for 1.7–2.0 folds (13). The ACCORD subset trials suggest that diabetic microvascular complications are correlated to the development of myocardial infarction (MI), stroke (14), and all-cause mortality (15).

However, the individual and combined effects of diabetic microvascular complications on stroke risk have not yet been fully elucidated. It is also elusive what the weight of each microvascular complication is on the development of stroke and whether it has an association with other common diabetic comorbidities. The answers of these questions can further guide diabetic management and stroke prevention. Current meta-analysis suggests multifactorial intensive intervention (blood pressure, glucose, cholesterol control) better reduces non-fatal stroke in diabetic patient (10). Therefore, we performed the present study to evaluate risks of ischemic and hemorrhagic strokes associated with microvascular complications in the DM population in Taiwan.

## Methods

### Data source

In this study, we utilized claims data of a DM patient-based data set selected from the whole insured population in Taiwan, obtained from the National Health Research Institutes (NHRI). All patient identifications are scrambled before releasing data to public to protect the patient privacy. Medical records of inpatients and outpatients from January 1, 1996 to December 31, 2011 are available for this study. The International Classification of Diseases, 9th Revision, Clinical Modification (ICD−9–CM) was used in disease diagnoses.

### Study subjects

For this study, we first used DM patient-based data set to identify patients with newly diagnosed stroke (ICD−9–CM: 430–435) from 2000 to 2011 among diabetic patients who had been prescribed medical care for type 2 DM (ICD−9–CM code: 250.x0 and 250.x2) for at least twice in one year. For each stroke case, we also randomly identified two control subjects from the DM patients without stroke, frequency matched by age (of every 5 years), sex, and diagnosis years of DM.

### Investigated variables and cofactors

Stroke events were classified into ischemic (ICD−9–CM: 433–435) and hemorrhagic (ICD−9–CM: 430–432) stroke. The investigated microvascular complications included nephropathy (NepP) (ICD−9–CM: 250.40–250.43 and 585–586), retinopathy (RetP) (CD−9–CM: 250.50–250.53, 362.01–362.06), and neuropathy (NeuP) (ICD-9-CM: 250.60–250.63, and 357.2). The baseline comorbidities were hypertension (ICD−9–CM: 401–405), hyperlipidemia (ICD−9–CM: 272), ischemic heart disease (IHD, ICD−9–CM: 410–414), and peripheral vascular disease (PVD, ICD−9–CM; 250.70–250.73, 440.20–440.24, and 440.29). These diagnoses appeared in the outpatient records for at least twice or in the inpatient records for at least once at baseline.

### Statistical analysis

Distributions of categorical demographic status and comorbidities were compared in the data analysis between stroke cases and controls, examined using the Chi-square test. Distributions of baseline prevalence rates of comorbidities among groups of patients with RetP, NeuP, and NepP and the combinations were compared and also examined using the Chi-square test. We used conditional logistic regression analysis to estimate the odds ratio (OR) and the 95% confidence interval (CI) of stroke associated with RetP, NeuP, and NepP and the combinations. Multivariable conditional logistic regression analyses were used controlling for age and sex, and co-morbidities to estimate adjusted OR (aOR). We used the statistical package of SAS version 9.4 (SAS Institute Inc. Cary, NC, USA) to perform all data analyses.

## Results

From the claims data for patients with DM, we identified 67,426 stroke cases and 134,852 patients without stroke as controls (Table 1). There were more men and the elderly. All co-morbidities were more prevalent in the stroke cases than in the controls. The prevalence of comorbidity increased with the number of microvascular complications (Table 2). Among the patients with three microvascular complications, 90.5% had hypertension, 57.9% had hyperlipidemia, 47.7% had IHD, and 31.7% had PVD.

**Table 1:** Distribution of demographics and comorbidities of diabetic patients with and without stroke in 2000-2011.

	**Non-stroke** ***N*** = **134,852**		**Stroke** ***N*** = **67,426**		
	***n***	**%**	***n***	**%**	***p*-value**
Sex					0.99
Women	56,406	41.8	28,203	41.8	
Men	78,446	58.2	39,223	58.2	
Age, year					0.99
20–49	11,444	8.49	5,722	8.49	
50–64	42,532	31.5	21,266	31.5	
65–79	59,200	43.9	29,600	43.9	
≥80	21,676	16.1	10,838	16.1	
**COMORBIDITY**					
Hypertension	67,743	50.2	48,980	72.6	<0.0001
Hyperlipidemia	46,276	34.3	26,857	39.8	<0.0001
IHD	25,706	19.1	19,354	28.7	<0.0001
PVD	5,501	4.08	4,725	7.01	<0.0001

*IHD, ischemic heart disease; PVD, peripheral vascular disease*.

**Table 2:** Comparison of comorbidity prevalence in diabetes patients by microangiopathy status.

**Variable (%)**	**None**	**NeuP**	**NepP**	**RetP**	**NeuP+ NepP**	**NeuP+ RetP**	**NepP+ RetP**	**NeuP+ NepP+RetP**	***p*-value**
**COMORBIDITY**									
Hypertension	52.8	69.3	73.4	69.1	82.8	75.3	85.9	90.5	<0.0001
Hyperlipidemia	32.9	47.5	40.9	47.4	57.7	58.2	53.1	57.9	<0.0001
IHD	19.0	28.9	33.7	24.3	43.6	31.1	37.3	47.7	<0.0001
PVD	3.11	11.0	7.15	7.99	19.8	19.7	15.2	31.7	<0.0001

*NepP, nephropathy; RetP, retinopathy; NeuP, neuropathy; IHD, ischemic heart disease; PVD, peripheral vascular disease*.

Table 3 shows that the stroke risk was associated with the individual and joint effects of NeuP, NepP, and RetP. In the multivariable model, the adjusted odds ratios (aORs) conferred by the unitary microvascular complication for stroke were 1.73 (95% CI = 1.66–1.79) for NeuP, 1.23 (95% CI = 1.19–1.27) for NepP, and 1.23 (95% CI = 1.19–1.27) for RetP. Further stroke subtype risk analysis showed significant estimated risk for ischemic stroke in association with NeuP (aOR = 1.77, 95% CI 1.70–1.85), NepP (aOR = 1.21, 95% CI 1.17–1.25), RetP (aOR = 1.53, 95% CI 1.42–1.64), and combination of microvascular complications. Likewise, hemorrhagic stroke had significant association with NeuP (aOR = 1.44, 95% CI 1.30–1.59), NepP (aOR = 1.34, 95% CI 1.23–1.46), RetP (aOR = 1.21, 95% CI 1.01–2.59), and combination of microvascular complications. Stroke risk was persistently associated with multiple microvascular complications. Patients with three microvascular complications had the highest risk of stroke in all models, and attained an aOR of 2.83 (95% CI = 2.58–3.09) for all stroke, after controlling for comorbidities. For patients with 3 microvascular complications. The corresponding aOR of ischemic stroke was 2.64 (95% CI 2.39–2.91) and that of hemorrhagic stroke was 4.12 (95% CI 3.25–5.22).

**Table 3:** Odds ratios by type of stroke and type of diabetic microangiopathy.

			**Crude**		**Multivariable**	
**Microangiopathy**	**Non-stroke**	**Stroke**	**OR**	**(95% CI)**	**aOR**	**(95% CI)**
**ALL STROKE**						
Total subject	134,852	67,426				
None	108,568	47,196	1.00	(reference)	1.00	(reference)
NeuP	7,632	6,098	1.97	(1.90–2.04)	1.73	(1.66–1.79)
NepP	11,586	6,958	1.46	(1.42–1.51)	1.23	(1.19–1.27)
RetP	2,275	1,544	1.66	(1.55–1.77)	1.47	(1.37–1.57)
NeuP+ NepP	2,182	2,516	3.02	(2.84–3.21)	2.25	(2.11–2.39)
NeuP+ RetP	670	738	2.86	(2.58–3.18)	2.34	(2.10–2.61)
NepP+ RetP	1,020	979	2.56	(2.34–2.80)	1.87	(1.71–2.05)
NeuP+ NepP+RetP	919	1,397	4.30	(3.94–4.69)	2.83	(2.58–3.09)
**ISCHEMIC STROKE**						
Total subject	113,514	56,757				
None	91,245	39,522	1.00	(reference)	1.00	(reference)
NeuP	6,428	5,323	2.04	(1.97–2.12)	1.77	(1.70–1.85)
NepP	9,855	5,821	1.45	(1.40–1.50)	1.21	(1.17–1.25)
RetP	1,900	1,339	1.73	(1.61–1.86)	1.53	(1.42–1.64)
NeuP+ NepP	1,858	2,183	3.09	(2.89–3.29)	2.26	(2.11–2.41)
NeuP+ RetP	565	646	2.98	(2.65–3.34)	2.39	(2.12–2.69)
NepP+ RetP	876	792	2.41	(2.18–2.66)	1.75	(1.58–1.94)
NeuP+ NepP+RetP	787	1,131	4.09	(3.72–4.50)	2.64	(2.39–2.91)
**HEMORRHAGIC STROKE**						
Total subject
None	17,323	7,674	1.00	(reference)	1.00	(reference)
NeuP	1,204	775	1.54	(1.39–1.70)	1.44	(1.30–1.59)
NepP	1,731	1,137	1.55	(1.42–1.68)	1.34	(1.23–1.46)
RetP	375	205	1.32	(1.10–1.58)	1.21	(1.01–1.46)
NeuP+ NepP	324	333	2.62	(2.22–3.09)	2.19	(1.85–2.59)
NeuP+ RetP	105	92	2.38	(1.77–3.21)	2.13	(1.57–2.88)
NepP+ RetP	144	187	3.40	(2.68–4.30)	2.60	(2.04–3.31)
NeuP+ NepP+RetP	132	266	5.61	(4.45–7.07)	4.12	(3.25–5.22)

*STROKE, cerebral vascular accident; NeuP, neuropathy; NepP, nephropathy, RetP, retinopathy; OR, odds ratio; and aOR, adjusted odds ratio compared to patients without microangiopathy; CI, confidence interval. Multivariable, Adjusted for hypertension, hyperlipidemia, ischemic heart disease, and peripheral vascular disease. Hemorrhagic stroke, ICD-9-CM, 430–432; Ischemic stroke, ICD-9-CM, 433–435*.

## Discussion

Our study showed that microvascular complications are significant stroke predictors, with a greater involvement for ischemic stroke than for hemorrhagic stroke. Combined multiple microvascular complications further increase the stroke risk. The findings of the present study are strengthened by its large sample size and statistic power from a nation-wide insurance claim data with the notions that the inevitable bias of the claim data such as misclassification and ethnic biases might weaken its generalizability.

It is generally accepted that the presence of microvascular complications reflects the increased severity of diabetes and should correlate with increased risk of stroke. A previous systemic review including 25 studies (*N* = 54117) has concluded that retinopathy and nephropathy were notable risk factors for cardiovascular diseases (13), but inconclusive for stroke. For example, some studies showed an increased risk of stroke in patients with diabetic retinopathy (14, 16) and nephropathy (17), whereas a Japanese study showed no difference of incidence of stroke between patients with and without diabetic microvascular complication (18). The present study confirms that all three common microvascular complications are independent risk factors for stroke. We further elucidate the combined effect of microvascular complications on ischemic and hemorrhagic stroke. The odds to have a stroke proportionately increased with the number of microvascular complication. It is justified to have such findings as previous studies suggesting similar pathological mechanisms of microangiopathy and macroangiopathy (11, 12).

In this study, we found both diabetic retinopathy and neuropathy are independent risk factor for ischemic stroke. Diabetic retinopathy is the most well-studied microvascular complication owing to the existence of direct objective measurement with visualized technologies (19). The finding of an increased risk of ischemic stroke in patients with diabetic retinopathy is consistent with previous studies (13, 16). A prospective study also suggests that diabetic retinopathy is an independent risk for small arterial stroke (20). It is believed that the change of retinal vasculature reflects the change of small vessels intracranially. However, due to the limitation of our data source, we cannot correlate the severity and types of retinopathy with the risk for stroke. Diabetic neuropathy was also found to be an independent risk factor for ischemic stroke even after taking into account of other conventional cardiovascular risk factors. Diabetic neuropathy is commonly associated with cardiac autonomic dysfunction (21) and can cause unstable baroreflex and cardiac rhythm (22). Although no direct evidence shows that diabetes-related cardiac dysautonomia leads to cerebral ischemia, studies have revealed that autonomic dysfunction by all causes is an independent risk of major cardiovascular events, including stroke (23). In addition, autonomic dysfunction contributes to atrial fibrillation (24). This role is not limited to favor atrial fibrillation occurrence but includes the possibility of determining atrial cardiopathy and subsequent ischemic strokes (25).

Although we found neither diabetic retinopathy nor neuropathy is associated with hemorrhagic stroke, we have a novel finding that reveals diabetic nephropathy has a stronger risk association with hemorrhagic stroke than with ischemic stroke. This phenomenon makes our finding about diabetic nephropathy particular interesting. We postulate that some of the common pathologies in patient with nephropathy contribute to this phenomenon. For example, the patients with diabetic nephropathy may predispose to hemorrhagic stroke because of platelet dysfunction and small vessel disease (26). The hemodynamic change may also play a role in hemorrhagic stroke. It has been noticed that chronic kidney disease (CKD) and cerebral vascular diseases often co-exist (27) because of the similarity of small vessels in the kidney and the brain (28). The perforating branches in brainstem and juxta-medullary afferent arterioles in kidney similarly receive blood directly from large arteries in order to ascertain sufficient perfusion to the vital organs (such as brainstem and nephrons). However, the wide pulse pressure “strains” these vessels when the arterial blood pressure is high (hypertension) and the endothelium of the small vessels are impaired (29) (e.g., diabetes). The weakening of these small vessels leads to decreased infiltration in the kidney and bleeding-prone microangiopathy in the brain (27). The hemodynamic change and microangiopathy are contributors of hemorrhagic stroke (30). In addition, arterial stiffness also has an important role in determining heamorrhagic stroke in these patients (31). The role of arterial stiffness is not limited to cerebral hemorrhage, but furthermore in ischemic stroke it may determine an altered cerebral microcirculation with failure of collateral circulation (32).

The positive correlation between diabetic nephropathy and ischemic stroke has been evidently concluded by a systemic review (17). In diabetic nephropathy, activation of renin-angiotensin system induces hypertension and stimulates NADPH oxidase. Hypertension is a known risk factor of cardiovascular diseases and NADPH oxidase further poses oxidative stress on the endothelium (33). CKD is also associated with dyslipidemia (34) and vascular calcification (35). All of these factors can result in an accelerated process of atherosclerosis and ischemic stroke.

Although we found the combination of microvascular complications persistently increased the risk of stroke, such increase was not proportional to the number of microvascular complications after adjusting for age, sex, and comorbidities. This finding implies the synergistic effect among these microvascular complications may not be independent of each other. Previous studies have found that one microvascular complication could predict the development of another microvascular complication (36) and these microvascular complications also share common pathogenesis (37). We found the number of microvascular complications positively correlated to the prevalence of comorbidities. Our findings illustrate a network between microvascular and macrovascular complications and microvascular complications have an important role in the development of stroke in diabetic patients. Taken together, this intriguing network among endothelial dysfunction, hyperglycemia, insulin resistance and chronic inflammation in the microcirculation (37) results in accelerated atherogenesis in large vesse (38).

## Strength and limitations

Findings of the present study are strengthened by the use of large population-based data with small selection bias. However, this study also has its limitations. Factors that may influence the risk of stroke such as personal lifestyle, and physical activity levels are unavailable in the database. Therefore, the associations of these variables to the risk of stroke in diabetes patients are not adjusted. Few patients with minor stroke symptoms might not be diagnosed and limited number of persons were selected into the control group by chance. Therefore, this misclassification may reduce little the measured stroke risk.

## Conclusion

Our study results support reliable relationships between microvascular complications and risk of stroke using the case-control analysis. Our data suggest a greater microvascular complication involvement for ischemic stroke than for hemorrhagic stroke. Higher risk is also noticed in patients with multiple microvascular complications. Identifying this high risk population is important for diabetes management and stroke prevention. Further study is needed to assess the benefit of intensive glycemic control and multifactorial intervention in this high risk population.

## Ethics statement

The Research Ethics Committee, China Medical University and Hospital, approved the use of the insurance claims data of Taiwan for research (CMUH104-REC2-115). Because all personal identifications had been changed into surrogate numbers before the data were released to users to protect privacy, no consent was required from study population. This study was conducted in accordance with the Declaration of Helsinki.

## Author contributions

WLC is the co-first author who conceptualized the research, drafted the manuscript, and analyzed the data. SFL, WLC, and FCS were responsible for conception and design of the work, analysis and interpretation of the data, writing and revision of the manuscript, quality assurance, and control. CHM, PCC, and SYC worked on the study design, acquired the data, analyzed and interpreted the data, and revised the manuscript. CHC and CLK analyzed and interpreted the data, reviewed the manuscript, made critical revision and suggestion of the manuscript for important intellectual content. All authors have read and approved the final manuscript.

### Conflict of interest statement

The authors declare that the research was conducted in the absence of any commercial or financial relationships that could be construed as a potential conflict of interest.
